# Purchases of medicines among community-dwelling older people: comparing people in the last 2 years of life and those who lived at least 2 years longer

**DOI:** 10.1007/s10433-019-00543-9

**Published:** 2019-11-14

**Authors:** Jutta Pulkki, Mari Aaltonen, Jani Raitanen, Pekka Rissanen, Marja Jylhä, Leena Forma

**Affiliations:** Faculty of Social Sciences, Tampere University, Tampere, Finland

**Keywords:** Purchases of medicines, Community-dwelling older people, Last years of life

## Abstract

While it is known that those who are living their last years are frequent users of social and health services, research about medicines at the end of life is scarce. We examined whether the proportions of purchasers and the types of prescription medicines purchased during a 2-year period differed between community-dwelling old people who died (decedents) in 2002, 2006 or 2011 and old people who lived at least 2 years longer (survivors) in Finland. We also examined how those differences changed over time. The study population was identified from nationwide registers and consisted of 174,097 community-dwelling people who were 70 years of age or older. Of these, 81,893 were decedents and 92,204 survivors. Data on purchases of medicines were gathered from the Finnish prescription database. Along with descriptive analyses, binary logistic regression analysis was used to find the association between decedent status and the purchase of medicines in general and different categories of medicines in particular. Almost all community-dwelling decedents and survivors purchased medicines at least once during the 2-year period. Over time, the proportion of purchasers increased in both groups but especially among survivors, thereby reducing the differences between the groups. However, the probability of purchasing medicines in general and different categories of medicine in particular remained significantly higher for decedents than for survivors after adjustments. This study shows that purchases of medication are concentrated at the end of life, as is the use of social and health services. However, the differences between decedents and survivors diminish over time.

## Introduction

The use of medicines among old people is very common and rapidly increasing (Barat et al. 2000; Crockett 2005; Fimea 2016). Medication has become commoner as the prevalence of diseases in old age has increased (Crimmins and Beltrán-Sánchez 2011), and diseases are more often treated with medicines (Giron et al. 1999).

The use of medicines differs between the oldest old and the youngest old (Gislason et al. 2005; Wastesson et al. 2012), between old men and old women (Bell et al. 2009; Gislason et al. 2005; Pokela et al. 2010) and between those living in different types of housing (Johnell and Fastbom 2012; Jyrkkä et al. 2006; Linjakumpu et al. 2002). Previous studies have also reported that old people at the end of life are frequent users of medicines (McNeil et al. 2016; Morin et al. 2017). While it is well known that those who are living their last years and months are frequent users of social and health services comparing to those who lived longer (Forma 2011; Lunney et al. 2007; Murphy and Martikainen 2013; Pot et al. 2009), the evidence on differences in medication is still scarce (Forma et al. 2007; Menec et al. 2007; Hoover et al. 2002).

Due to multimorbidity, old people at the end of life are particularly at risk of polypharmacy and related adverse effects (Cruz-Jentoft et al. 2012; Paque et al. 2019a; Holmes et al. 2006). There is no consistent definition for polypharmacy, and especially it is not clear when it is appropriate and when inappropriate (Mortazavi et al. 2016). For example, in outpatient settings, the number of four medicines has often been used as a dividing line for polypharmacy (Rollason and Vogt 2003), referring to multiple use of medicines but not necessary on inappropriate medication. It has been suggested that the otherwise appropriate medication of frail old people, especially those with limited life expectancy, should be reconsidered and unnecessary medicines are deprescribed (Holmes et al. 2006; Paque et al. 2019a). However, this is not often the case in practice, and numerous studies have demonstrated the frequent use of unnecessary and inappropriate medication among old people near death (Maddison et al. 2011; Curtin et al. 2018; Paque et al. 2019b).

Polypharmacy, especially the inappropriate medicine usage, is not just a disadvantage at the individual level, but increases also the public costs of medicines in countries with inclusive public welfare systems. In Finland, for example, the health insurance scheme covers a high proportion of medicine expenditures for all permanent residents. Reimbursements are paid for almost all medicines prescribed by medical doctors and purchased by community-dwelling people, e.g. those who live at home or in service houses. Ninety-five per cent of those aged 65 or older obtained reimbursements in 2015, and reimbursements were commoner among the oldest old than the younger old (Fimea 2016).

Among old people in Finland, the living in institutions such as nursing homes has dramatically decreased and the living in community settings, especially in service houses with 24-h care, has steadily increased, since the beginning of the twenty-first century (National Institute for Health and Welfare 2017). Institutional care and community care facilities serve similar care needs, but their financing differs. First, in institutions, all costs including medicines are included in the monthly care fee, and purchases of medicines are not shown on national registers. In service houses, on the other hand, residents are considered to be home-dwelling and they need to pay separately for everything themselves, including rent, care services, food, medicines, etc. This shift from all-inclusive institutional care to community care where old people purchase medicines themselves has resulted in growing proportions of purchasers and increased reimbursements for prescribed medicines in Finland (Aaltonen et al. 2016; Blomgren and Einiö 2015).

This study focused on the proportion of purchasers and the types of prescribed medicine purchased by community-dwelling old people at the end of life and by those who lived at least 2 years longer. It also focused on how those purchases changed over time. The results reveal increased trends in old people’s purchases of medicines in an era when community care arrangements have become considerably commoner in Finland. Purchases of medicine were studied for the 2 years prior to death among those who died (decedents) at the age of 70 or older in the years 2002, 2006 and 2011, and for the same period among old people who lived for at least 2 years after 2002, 2006 and 2011 (survivors). The detailed research questions were as follows: (1) How did the proportions of purchasers and the types of medicine purchased differ between decedents and survivors? (2) How did these differences change between the years 2002, 2006 and 2011?

## Materials and methods

### Study population

This study is part of a larger project called ‘New dynamic of longevity and the changing needs for services’ (COCTEL). The original COCTEL data include all those who died in Finland at the age of 70 years or older in 2002–2013. Decedents were identified from the nationwide Causes of Death Register (maintained by Statistics Finland). For almost every decedent, a surviving counterpart, i.e. a person who lived at least 2 years longer, was identified from the Finnish Population Information System (maintained by Statistics Finland), using 40% random samples of persons born in the same year as the decedent. In the original COCTEL data, the survivors are individually matched with corresponding decedents according to age, gender and municipality of residence. For more details on the COCTEL study design, see Forma (2011).

In this study, the groups of decedents for the years 2002, 2006 and 2011 were compared with the groups of survivors for each year. In the original COCTEL data, the number of decedents and survivors was 29,337 in 2002, 32,140 in 2006 and 34,036 in 2011. Community-dwelling people were identified among these and selected for this study, since purchases of medicines by people living in institutions are not registered. The identification was made using data from the Care Register for Healthcare and the Care Register for Social Welfare (both maintained by the National Institute for Health and Welfare), which are linked to each person in the COCTEL data. First, as the register data do not explicitly count days at home, old people who had no registered days in any social or healthcare institution were considered to be living at home. Second, people living in service housing with 24-h care were considered to be community-dwelling, as they buy their own medicines. All those who lived for at least 90 of the 730 days at home or in service housing during the 2-year study period were included in the study population. It was probable that people would have purchased any necessary medicines within those 90 days, as many medicine prescriptions are for three months.

Appendix Table 5 shows the proportions of community-dwelling decedents and survivors in the original COCTEL data set. Because of the inclusion of community dwellers only, the matched pairs were no longer accurate, and the comparisons were made at group level instead of individual level.

Permission to use individualized data was granted to COCTEL by the registers’ administrators, and the study plans were approved by the Pirkanmaa Hospital District Ethics Committee.

### Data on purchases of medicines

Purchases of prescribed medicines are recorded on national registers in Finland as well as in other Nordic countries, and these registers are often used as data sources in medicine usage studies (Bell et al. 2009; Gislason et al. 2005; Johnell and Fastbom 2012; Wastesson et al. 2012; Morin et al. 2017). In this study, data regarding the date and type [using WHOCC (2013) five-level Anatomical Therapeutic Chemical (ATC) codes] of each purchase of medicine were collected from the National Prescription Register maintained by the Social Insurance Institution. Information on each person’s purchases of prescribed medicines was collected for a 2-year period: for decedents, 2 years (730 days) prior to death; for survivors, for the same calendar days as their decedent counterpart. Data from the National Prescription Register and other registers were linked using the unique individual identification code possessed by every Finnish resident.

Each purchase of medicine was categorized as belonging to one of the 14 main ATC categories. Dichotomous variable was created to describe whether a person had purchased any medicines at least once during the 2-year period (0 = no purchases, 1 = at least one purchase), and whether a person had purchased separate medicines in each of the 14 main categories. Dummy variables indicating purchases in these main categories were calculated together to reveal how many ATC categories of decedents and survivors had bought medicines within the 2 years. This variable could have values from 0 to 14. A dichotomous outcome was created from this variable by dividing it to those who purchased medicines from less than four, or at least four ATC categories to indicate polypharmacy. This means that people have purchased at least four different medicines but maybe even more, if they have bought more than one item of drugs from one ATC category.

### Data on days in a community-dwelling setting

Each person’s days spent in community-dwelling settings, i.e. at home or in service housing with 24-hour care, were calculated on the basis of admission and discharge dates recorded in the Care Register for Healthcare and the Care Register for Social Welfare. The continuous variable ‘the number of days in community settings’ was created, to identify community dwellers and adjust the length of stay in the community, where medicine purchases could take place. The adjustment was needed because it is known that decedents have more days in institutions where medication is included in the care, and because the days in community-dwelling settings increased significantly within the study period, thereby also increasing the opportunities to buy medicines.

### Analysis

To analyse the differences between decedents and survivors in the different years, the proportions of purchasers of medicines were analysed, both in total and separately for each ATC category, by using cross-tabulations, chi-square tests and rate ratios. An independent sample *t* test was used to compare the differences in means of medicine purchases in several ATC categories. Analyses were conducted separately for the years 2002, 2006 and 2011, for men and women, and for different age groups (70–79, 80–89 and at least 90 years).

Binary logistic regression models were used to compare the likelihood of the purchase of medicines in total, purchases in at least four ATC categories, and purchases in the commonest ATC categories, among decedents and survivors. The models were run stepwise: in crude models, status (decedent versus survivor) was used as an independent variable; in model 1, status, age, gender, year (of death), the number of days in community-dwelling settings and the interaction term (status*year) were used. The interaction term was used to examine whether the effect of decedent status changed over time. If the p value was less than 0.001, it was concluded that the difference was significant. The analyses were performed with SPSS (v.22) statistical software.

## Results

### Description

The study population comprised in total 174,097 community-dwelling old people, and the proportion of community dwellers increased between the study periods. The mean age of the study population increased too, especially the proportion of people aged 90 and older (Table 1). The mean number of days lived in the community increased steadily between the study periods among decedents, whereas survivors showed only minor changes.

**Table 1:** Description of the study population

	2002			2006			2011		
	Decedents	Survivors	Total	Decedents	Survivors	Total	Decedents	Survivors	Total
	*n* = 24,797	*n* = 28,285	*n* = 53,082	*n* = 27,187	*n* = 30,675	*n* = 57,862	*n* = 29,909	*n* = 33,244	*n* = 63,153
Men (%)	45.5	43.2	44.3	46.4	44.2	45.3	46.2	44.8	45.4
Mean age	80.3	80.7	80.5	82.0	82.3	82.1	82.7	83.0	82.8
Age groups (%)									
70–79	46.5	43.9	45.1	37.1	35.2	36.1	32.3	30.8	31.5
80–89	47.1	48.8	48.0	48.5	49.4	49.0	51.5	51.7	51.6
At least 90	6.4	7.3	6.9	14.3	15.4	14.9	16.2	17.5	16.9
Days in community-dwelling settings									
Mean (SD)	615.5 (148.1)	710.9 (68.4)	666.4 (122.5)	619.5 (145.4)	707.0 (77.1)	665.9 (122.5)	632.6 (132.9)	711.9 (65.4)	674.4 (110.4)
Median	675.0	730.0	720.0	678.0	729.0	718.0	684.0	730.0	721.0

### Purchasers of medicines

Altogether, 93–98% of community-dwelling old people purchased prescribed medicines within the 2-year period, with an increase from 2002 to 2011. While decedents bought medicines more frequently than survivors in 2002, the difference diminished in 2006, and in 2011 survivors purchased medicines slightly more frequently than decedents. This trend was shown in different age groups. Even though the differences in total purchases between decedents and survivors were statistically significant, absolute differences were very small (rate ratios (RR) = 1.03, 1.02 and 1.00, separately) (Table 2).

**Table 2:** Proportion (%) of purchasers of medicines and rate ratios (RR) of the proportions during the 2-year period among community-dwelling decedents (*D*) and survivors (*S*): total, by the ATC categories and in the years 2002, 2006 and 2011

		2002				2006				2011			
		*D*	*S*	*P* value^a^	RR	*D*	*S*	*P* value^a^	RR	*D*	*S*	*P* value^a^	RR
Total		95.4	92.8	< .001	1.03	96.2	94.1	< .001	1.02	97.5	97.9	< .001	1.00
Gender													
Men		94.6	90.8	< .001	1.04	95.8	92.5	< .001	1.04	97.4	97.5	.603	1.00
Women		95.4	92.8	< .001	1.03	96.2	94.1	< .001	1.02	97.5	97.9	< .001	1.00
Age group													
70–79		95.0	90.5	< .001	1.05	95.5	91.7	< .001	1.04	96.9	97.1	.429	1.00
80–89		95.8	94.5	< .001	1.01	96.9	95.4	< .001	1.02	97.8	98.4	< .001	0.99
At least 90		95.2	95.2	1.00	1.00	96.0	95.4	.136	1.01	97.5	98.2	< .05	0.99
ATC categories^b^													
A	Alimentary tract/metabolism	61.5	39.4	< .001	1.56	65.3	43.4	< .001	1.50	74.9	58.0	< .001	1.29
B	Blood/blood forming organs	32.2	20.0	< .001	1.61	38.4	25.0	< .001	1.54	48.9	35.4	< .001	1.38
C	Cardiovascular	81.0	70.6	< .001	1.15	83.9	77.1	< .001	1.09	87.5	84.8	< .001	1.03
D	Dermatologicals	21.4	18.8	< .001	1.14	17.3	15.9	< .001	1.09	17.0	19.1	< .001	0.89
G	Genito-urinary systems/sex horm.	23.2	22.2	< .05	1.05	25.0	24.2	< .05	1.03	27.6	31.0	< .001	0.89
H	Systemic hormones	28.2	16.1	< .001	1.75	28.9	16.8	< .001	1.72	35.5	23.2	< .001	1.53
J	Anti-infectives for systemic use	62.3	47.3	< .001	1.32	60.9	42.9	< .001	1.42	67.7	57.8	< .001	1.17
L	Antineoplastic/immunomodulating agents	8.4	2.2	< .001	3.82	10.5	3.5	< .001	3.00	12.6	5.1	< .001	2.47
M	Musculoskeletal system	53.4	47.4	< .001	1.13	48.2	44.3	< .001	1.09	41.5	43.8	< .001	0.95
N	Central nervous system	65.8	41.5	< .001	1.59	68.6	47.2	< .001	1.45	83.5	69.9	< .001	1.19
P	Antiparasitic products	2.3	1.2	< .001	1.92	3.3	1.6	< .001	2.06	4.6	3.0	< .001	1.53
R	Respiratory system	33.9	30.0	< .001	1.13	26.8	19.8	< .001	1.35	27.4	23.6	< .001	1.16
S	Sensory organs	18.3	20.6	< .001	0.89	19.4	21.5	< .001	0.90	22.0	27.7	< .001	0.79
ATC categories^c^ in total													
Mean (median)		4.96 (5)	3.77 (4)	< .001		4.97 (5)	3.82 (4)	< .001		5.51 (5)	4.82 (5)	< .001	

^a^Pearson’s chi-square tests

^b^The number of purchasers of medicines belonging to category ‘various’ (V) was so low that the results are not presented here

^c^Independent samples t test

Also in logistic regression model 1, the interaction term (status*year) indicates that the difference between decedents’ and survivors’ total purchases of medicines fell from 2002 to 2011 [odds ratio (OR) = 0.48)]. However, decedents still bought medicines slightly more often after adjustment for age, gender and the number of days in community settings (in 2002 OR = 2.10; in 2011 OR = 2.10*0.48 = 1.01) (Table 3).

**Table 3:** Purchases of medicines among decedents and survivors—in total, and from four or more ACT categories; odd ratios from logistic regression models

	Purchases in total		≥ 4 ATC	
	Crude^a^	Model 1^b^	Crude^a^	Model 1^b^
Status (surv = ref)	**1.40**	**2.10**	**2.22**	**2.46**
Year (2002 = ref)				
2006		**1.19**		**1.06**
2011		**3.45**		**2.27**
Status*year (2002 = ref)				
2006		0.97		1.01
2011		**0.48**		**0.74**

^a^Crude = only independent variable was decedent status

^b^Model 1 = crude + adjusted for age, gender, year, days at community-dwelling setting, and interaction between decedent status and year

Sig < 0.001—bolded

### Purchases in several ATC categories

**Fig. 1:**
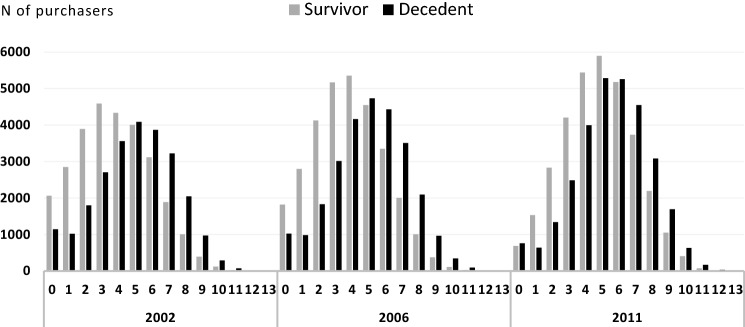
Total number of ATC categories (min 0, max 14) from which survivors and decedents bought medicines within 2 years preceding the years 2002, 2006 and 2011

Medicines from four or larger number of ATC categories were purchased more often during the latest study period than during earlier years, by both decedents and survivors. Figure 1 shows that the decedents bought medicines from five and more ATC categories more often than survivors within the 2 years preceding the years 2002 and 2006, and six and more ATC categories in year 2011. The shift over time was seen more clearly among those who lived at least 2 years longer, reducing the difference between decedents and survivors (Fig. 1, Table 2).

The adjusted results showed also that the difference between the status groups in medicines purchased from at least four ATC categories diminished comparing the year 2011 to 2002 (status*year OR = 0.74). However, in 2011 decedents still bought medicines from at least four ATC categories more often than did survivors (OR = 2.47*0.74 = 1.83) (Table 3).

### Purchases by ATC category

Decedents purchased medicines in most of the ATC categories significantly more commonly than did survivors, but the differences diminished over time (RRs, Table 2). Both decedents and survivors most commonly purchased medicines belonging to the ‘cardiovascular’ (C) category. In addition, purchases in the categories ‘central nervous system’ (N, including for example medication for Alzheimer’s disease), ‘alimentary tract’ (A, including drugs used in diabetes), ‘anti-infectives’ (J, including drugs used in infections) and ‘musculoskeletal system’ (M, including muscle relaxants and painkillers) were common in both groups. The frequency of buying medicines increased from 2002 to 2011 in all of these categories except M (Table 2).

The differences between decedents and survivors in purchases of medicines in the five most common categories (A, C, J, M and N) diminished from 2002 to 2011 after adjustments for age, gender and the number of days in community (ORs for interaction term status*year > 1, separately). However, in 2011 decedents still bought medicines more often than survivors in all categories (for A OR = 2.36*0.84 = 1.98; C OR = 1.37; J OR = 1.56; N OR = 1.91) other than M (OR = 1.35*0.71 = 0.96) (Table 4).

**Table 4:** Purchases of medicines among decedents and survivors by the most common ACT categories, odds ratios from logistic regression models

	A^a^		C^b^		J^c^		M^d^		N^e^	
	Crude^f^	Model 1^g^	Crude^f^	Model 1^g^	Crude^f^	Model 1^g^	Crude^f^	Model 1^g^	Crude^f^	Model 1^g^
Status (surv = ref)	**1.26**	**2.36**	**1.53**	**1.99**	**1.79**	**1.88**	**1.10**	**1.35**	**2.36**	**2.33**
Year (2002 = ref)										
2006		**1.18**		**1.34**		**0.83**		**0.90**		**1.21**
2011		**2.15**		**2.18**		**1.51**		**0.89**		**3.20**
Status* year (2002 = ref)										
2006		1.00		**0.86**		**1.12**		**0.92**		*0.92*
2011		**0.84**		**0.69**		**0.83**		**0.71**		**0.82**

^a^A = alimentary tract/metabolism

^b^C = cardiovascular

^c^J = anti-infectives for systemic use

^d^M = musculoskeletal system

^e^N = central nervous system

^f^ Crude = only independent variable was decedent status

^g^ Model 1 = crude + adjusted for age, gender, year, days at community-dwelling setting and interaction between decedent status and year

Sig < 0.001—bolded, Sig < 0.05—italicized

## Discussion

Almost all community-dwelling old people purchased medicines within the 2-year study periods. The proportion of purchasers of medicines increased among both decedents and survivors from 2002 to 2011, which is in line with the general trend of increasing prescription numbers and medicine usage among old people in Finland (Jyrkkä et al. 2006; Linjakumpu et al. 2002). During the study period, the shift from institutions to community care has been dramatic in Finland (National Institute for Health and Welfare 2017) and is shown also in increased purchases of prescribed medicines (see Blomgren and Einiö 2015). Our results reflect this shift too.

Our results show that not only those community-dwelling old people who lived their last years (McNeil et al. 2016; Morin et al. 2017) but also those who were surviving longer become more frequent purchasers of medicines. The differences in proportions of purchasers in total and in different ATC groups reduced between decedents and survivors during the study period. Based on our analysis, we cannot give a full explanation of why the differences between the groups diminished. We can say, though, that as age was controlled for in our analysis, the fact that people in our study population—especially survivors—were older year by year does not explain the decrease in the differences. The proportion of the oldest old was higher among survivors than among decedents, because the oldest old near death more frequently live in institutions (see Appendix Table 5). The results might have changed, although probably not dramatically, if our data had included purchases made by the most ill oldest old who were living their last years in institutions.

While decedents bought medicines more commonly than survivors in almost all ATC categories, survivors were more likely to purchase medicines in ATC categories for genito-urinary systems and sensory organs, i.e. for less severe illnesses. That is, at least some of the differences may be attributable to the different diseases and the severity of these. The shift in long-term care arrangements in Finland has most probably changed health conditions and the need for medicines of old people living in the community. Thus, people living in the community in 2011 compared with 2002 were not just older (Appendix Table 5) but probably also had different health status, which may explain the changes in purchases of medicines in both groups. However, we can only speculate this as neither the health status nor the indications for the medications were available for the community dwellers in our data.

The increased use of medicines may also reflect changing trends in healthcare practices. In our study, however, the study period was relatively short, while most of the changes in diagnostic or more active care have occurred over a longer time span (Crimmins and Beltrán-Sánchez 2011). Instead, the changes in Finnish reimbursement policies are known to have influenced medication purchases, as well as prescription practices at short notice. For example, purchases of medicines for Alzheimer’s disease (ATC codes in the N06D category) are increasing, partly because these medicines are increasingly reimbursed out of national health insurance and their prices declined soon after their approval by the subsidies programme (Aaltonen et al. 2016). In addition, some medicines—for example, those in the musculoskeletal category—can increasingly be bought over the counter without a prescription and are therefore not shown in national registers. These kinds of change in reimbursement and other criteria were made to numerous medicines in Finland during the study period, and they may explain some of the temporal changes in this study.

Medicines in the cardiovascular, central nervous system, alimentary tract and anti-infectives categories were those most commonly purchased by community-dwelling old people in their last years of life. Medicines in these categories, except anti-infectives, have been found to be the most commonly used among old people in several studies (Barat et al. 2000; Giron et al. 1999; Linjakumpu et al. 2002). The proportion of those buying medicines for cardiovascular diseases at the end of life was relatively high compared with other studies (Barat et al. 2000; Giron et al. 1999; Mizokami et al. 2012), but was similar to numbers reported in previous Finnish studies (Jyrkkä et al. 2006; Linjakumpu et al. 2002). This might be a result of differences in the data rather than in clinical practices between countries.

It has been found that the inappropriate use of medicines is commoner in community settings, where medication is not carefully supervised than it is in nursing homes staffed by healthcare professionals (Elliot 2006; Lane et al. 2004). Our purpose was not to go into the details of whether medication is inappropriate, but to show the trends of polypharmacy, which may also indicate the inappropriate medication. Our results show that purchases of medicines from several ATC categories, i.e. polypharmacy, increased, a trend that has also been shown in previous studies (Hajjar et al. 2007; Haider et al. 2007; Hovstadius et al. 2010). Our results highlight the need to monitor and reconsider the medication of the increasing number of community-dwelling old people, in order to reduce the risk of adverse effects related to inappropriate medication, particularly when life expectancy is limited (Lane et al. 2004; Holmes et al. 2006; Maddison et al. 2011).

A few limitations need to be addressed concerning the data. It is likely that people in the survivors group in earlier years may be survivors or decedents in later years. As our research data did not allow to take this possible overlapping into account, this may potentially threaten the internal validity of the study. Overlapping may decrease the standard error and thus increase statistically significant findings. However, we surmise that the overlapping is minor as the survivors were identified from the 40% random sample, they may have died in a different year than studied, or they may not live in the community anymore. Also, in the case of overlapping, people’s situations may have altered so rapidly, as at old age they often do, that the effects on the results are indifferent.

The Finnish Prescription Register includes information on all medicine purchases reimbursed by the health insurance scheme, but only for residents living in non-institutional settings. Therefore, our findings only apply to those who were living at home or in service housing. Additionally, the Prescription Register includes only prescription medicines, not over-the-counter medicine purchases. For purchases of prescribed medicines, Finland’s administrative registers have been found to be reliable and comprehensive (Gissler and Haukka 2004). Therefore, in our understanding, our study reliably describes the trends in purchases of medicines among community-dwelling old people. However, such purchases do not reveal whether the medicines are actually used; to study this, methods other than registers are needed (Barat et al. 2000; Linjakumpu et al. 2002; Mizokami et al. 2012).

## Conclusions

This study shows the growing proportion of medication purchasers among community-dwelling old people, along with increasing longevity and the trend of living in the community. Community-dwelling old people in their last 2 years of life purchased medicines more frequently than those who lived at least 2 years longer, but the differences decreased over time. The reasons for the diminished differences and whether the differences between these two groups have changed further in more recent years are yet to be investigated.

## Appendix

See Table 5.

**Table 5:** Proportions of community-dwelling people of the original COCTEL dataset—decedents and survivors in years 2002, 2006 and 2011, by gender and age (*n* = for total COCTEL dataset)

	2002		2006		2011	
	Decedents	Survivors	Decedents	Survivors	Decedents	Survivors
COCTEL dataset	*n *= 29,337	*n *= 29,337	*n *= 32,140	*n *= 32,140	*n *= 34,036	*n *= 34,036
% of all	84.5	96.4	84.6	95.4	87.9	97.7
Gender (%)						
Men	90.5	98.2	90.9	97.7	91.8	98.8
Women	80.1	95.1	79.8	93.7	84.7	96.8
Age group (%)						
70–79	91.6	98.8	92.5	98.9	93.7	99.4
80–89	81.1	95.7	83.3	95.7	87.6	97.8
At least 90	67.8	87.9	72.4	87.7	78.9	94.4
